# Regulation of γδT17 cells by *Mycobacterium vaccae* through interference with Notch/Jagged1 signaling pathway

**DOI:** 10.1590/1414-431X20209551

**Published:** 2020-10-07

**Authors:** Yi En Yao, Jing Hong Zhang, Xiao Ju Chen, Jian Lin Huang, Qi Xiang Sun, Wei Wei Liu, Huan Zeng, Chao Qian Li

**Affiliations:** 1 Department of Respiratory Medicine, the First Affiliated Hospital of Guangxi Medical University, Nanning, Guangxi, China; 2 The Second Affiliated Hospital, Guangxi Medical University, Nanning, Guangxi, China; 3 Department of Internal Medicine, Affiliated Tumor Hospital, Guangxi Medical University, Nanning, Guangxi, China; 4 Department of Critical Care, First People's Hospital of Yulin City, Nanning, Guangxi, China; 5 Department of Emergency Medicine, the First Affiliated Hospital of Guangxi Medical University, Nanning, Guangxi, China

**Keywords:** Asthma, Mycobacterium vaccae, Jagged1 receptor, γδT17, Notch signaling

## Abstract

The objective of this study was to investigate the effect of *Mycobacterium vaccae* on Jagged 1 and gamma delta T17 (γδT17) cells in asthmatic mice. An asthma mouse model was established through immunization with ovalbumin (OVA). Gamma-secretase inhibitor (DAPT) was used to block the Notch signaling pathway. *M. vaccae* was used to treat asthma, and related indicators were measured. Blocking Notch signaling inhibited the production of γδT17 cells and secretion of cytokine interleukin (IL)-17, which was accompanied by a decrease in Jagged1 mRNA and protein expression in the treated asthma group compared with the untreated asthma group. Similarly, treatment with *M. vaccae* inhibited Jagged1 expression and γδT17 cell production, which was associated with decreased airway inflammation and reactivity. The Notch signaling pathway may play a role in the pathogenesis of asthma through the induction of Jagged1 receptor. On the other hand, the inhibitory effect of *M. vaccae* on Jagged1 receptor in γδT17 cells could be used for the prevention and treatment of asthma.

## Introduction

Bronchial asthma, or simply asthma, is a chronic inflammatory disease of the airways that involves the coordination of various cells including eosinophils, T lymphocytes, mast cells, and neutrophils (1). Despite the progression of asthma-based research, the mechanisms of asthma pathogenesis and progression have not been fully elucidated.

The immunological mechanisms involved in asthma progression have attracted lot of attention over the past decade. One of the classical immunological mechanisms recognized in the world is the Th1/Th2 imbalance (2), in which, the function of Th1 cells is insufficient, and Th2 cells are hyperactive. In this regard, gamma delta T (γδT) cells can play a key role in the regulation of allergic inflammation in the airways. γδT are immune cells that have distinctive T-cell receptors (TCR) on their surface, TCR-γ and TCR-δ, and play an important role in innate and adaptive immune responses (3). Treatment of mice with anti-γδ TCR antibodies has been shown to increase serum levels of IgE, which is known to play a critical role in asthma pathogenesis (4). In addition, γδT cells are an important source of interleukin (IL)-17 (5), which is involved in the recruitment of concentrated granulocytes and participates in the development of various inflammatory diseases (6,7).

IL-17 has been shown to be positively correlated with asthma severity. IL-17 plays a role in regulating inflammation, responsiveness, and airway remodeling (8,9). In addition, it could be a target for gene therapy in hormone-resistant asthma patients (10). In fact, γδT17 is a subpopulation of γδT cells that express IL-17 at higher rates, compared to other T lymphocytes (11). Thus, it is necessary to develop new strategies for the treatment of bronchial asthma. In this context, we recently showed that inhaling *Mycobacterium phlei* reduces lung inflammation in asthma by restoring the balance of γδT and Th1/Th2 cells (12,13). γδT cells can promote airway inflammation and airway hyperresponsiveness by secreting a large arsenal of pro-inflammatory cytokines that can modulate Th2-mediated pulmonary inflammation (14).

The mechanism of *M. phlei* that regulates γδT17 cells in asthma is not clear. Notch signaling pathway, the main factor that controls lymphocyte activation and differentiation, has been shown to be related to IL-17 (15). Notch signaling pathway plays an important role in the development of asthma by inducing Th2 cell differentiation (16) and may actively maintain a balance of cell types (17). Notch ligand Jagged1 has been shown to be highly expressed in the proximal asthmatic airways, which may lead to enhanced Th2 cell development (18,19). Many previous findings indicate that it may be possible to treat allergic asthma by inhibiting the production of Th2 and Th17 cytokines and downregulating the expression of Jagged1 (20). Consequently, the mechanism of action of the Notch signaling pathway in asthma has been intensely studied in recent years. However, the role of Notch signaling in the development of γδT17 cells in the treatment of asthma using *M. vaccae* is still poorly understood. In this regard, gamma-secretase inhibitor (DAPT) of Notch signaling can regulate Jagged1-Hes1 signaling in mice by blocking the Notch signaling pathway (21). In this work, we used DAPT in an ovalbumin (OVA) asthma model to study the effects of Jagged1 on γδT17 cells during asthmatic inflammation. In addition, we investigated the effect of aerosolized *M. vaccae* on the Jagged1-induced γδT17 inflammatory response in an asthmatic mouse model.

## Materials and Methods

### Materials

The materials used were: ovalbumin (OVA) reagent (Sigma, USA); phorbol-myristate actetate (PMA, Sigma); ionomycin (Sigma); aluminum hydroxide gel (Pierce, USA); *Mycobacterium vaccae* FU22.5 Ug injection (Anhui Zhifeilong Kema Bio-Pharmaceutical Co., Ltd., China); DATP (Selleck, USA), mouse IL-17A ELISA kit (MultiSciences: Wuhan McGee Biotechnology Co., Ltd., China); TRIzol reagent, reverse transcription kit, SYBR Green nucleic acid dye, PCR primer design and synthesis (TakalaRa, Japan); Jagged1 immunohistochemical antibody (CST, USA); flow antibody PerCP-Cy5-5 CD3, APC γδT, PE IL-17 (American eBioscience, USA); antibody monensin, fixed/membrane-breaking liquid (BD Co., USA); Finepointe NAM Mouse Noninvasive Lung Function Tester (BUXCO, USA).

### Animals and experimental groups

Healthy male C57 mice (4-6 weeks old; weight 20±2 g) were provided by the Animal Experimental Center of Guangxi Medical University (China) and housed in pathogen-free cages under SPF laboratory conditions. They were maintained in an air-conditioned room with a suitable temperature and humidity, and free access to food and water. Ethical approval was obtained from Guangxi Medical University Animal Ethics Committee before the study started.

Mice were randomly distributed into five groups containing 8 mice each: control group, asthma group (OVA), blocking group (DAPT + OVA), prevention group (*M. vaccae* + OVA), and treatment group (OVA + *M. vaccae*).

### Asthma model, prevention, and treatment methods

The asthma model mice were sensitized by treatment with 25 µg of OVA emulsified in aluminum hydroxide gel (1 mg) on days 0, 7, and 14. The animals were then challenged with 2% OVA diluted in phosphate-buffered saline (PBS) for 30 min on days 21-28. For the blocking group, the γ-secretase inhibitor DAPT (0.3 mg/kg) was inhaled for 30 min before the OVA treatment. Furthermore, 22.5 µg of the *M. vaccae* powder was dissolved in 10 mL of physiological saline and was given to mice by inhalation on days 21-28 for the prevention group, and on days 28-35 for the treatment group. In the control group, each of the above-mentioned treatments were replaced with the same amount of normal saline. Similarly, treatments for the asthma group were replaced with the same amount of normal saline. Mice were sacrificed by intraperitoneal injection of 10% chloral hydrate (0.1 mL), and specimens were collected within 24 h of the last treatment.

### Mouse airway reactivity test

The mice were placed in test chambers and the ventilator was turned on to detect the specific airway resistance (sRaw) triggered by PBS and different methacholine (Mch) concentrations (6.25, 12.5, 25, and 50 mg/mL).

### Lung histopathology

The left lung tissue was fixed with 4% paraformaldehyde for 24 h, rinsed, dehydrated in ethanol gradient, and embedded in paraffin. The paraffin sections were then sliced at a thickness of 4 μm and stained with hematoxylin and eosin (HE) and with Periodic acid-Schiff (PAS). The sections were observed under a light microscope (Olympus, Japan).

Then, 4-mm paraffin-embedded tissue sections were dewaxed in xylene and rehydrated in graded alcohols. Endogenous peroxidase was blocked using 3% hydrogen peroxide. Antigen recovery was performed by boiling tissue sections in 10 mM citrate buffer (pH 6.0) for 10 min. The tissue sections were incubated in 5% goat serum albumin and subsequently in polyclonal goat anti-human Jagged1 antibody (1:2500; D4Y1R, CST) for 24 h at 4°C,Then, the tissue sections were incubated with the horseradish peroxidase-conjugated secondary antibody for 60 min at room temperature, and subsequently stained with 3,3-diaminobenzidine and counterstained with hematoxylin blue. Negative controls were performed by replacing the primary antibody with unimmunized serum. Control tissue sections were treated in parallel with the samples. All tissue sections were observed under an optical microscope (Olympus).

The proteins were detected by immunofluorescence assay. For Jagged1 staining, air-dried cryostat sections of lung tissue (2 mm) were fixed using ice-cold methanol for 20 min, air-dried again, incubated in PBS containing 20% fetal calf serum (FCS) for 10 min, and exposed to 30 mL of 1.5% H_2_O_2_ for cell permeabilization. Then, the sections were washed twice with PBS and incubated with an avidin D solution for 10 min. Endogenous peroxidase was blocked using a biotin solution to inhibit endogenous biotin, washed again, and subsequently incubated with rabbit anti-mouse Jagged1 antibody (1:1000; D4Y1R, CST) overnight at 4°C. Next, the sections were washed twice with PBS, treated with the goat anti-mouse IgG secondary antibodies for 2 h in the dark. Finally, the sections were washed and observed by fluorescence microscopy (Olympus).

### ELISA test

Mice were anesthetized with 10% chloral hydrate, and blood was obtained from the eyeballs. The collected blood was allowed to stand for 3 h at 25°C and centrifuged at 700 *g* for 10 min. The supernatant was collected and stored at -80°C. The serum concentrations of IL-17 in mice in each group were measured by commercial ELISA kits according to the manufacturer's instructions (MultiSciences: Wuhan McGee Biotechnology Co., Ltd.). The IL-17 ELISA kits detect from 47 to 3000 pg/mL. Absorbance values at 450 nm were obtained by a micro-plate ELISA reader (BioTek, USA). All samples were analyzed in duplicate and IL-17 concentrations were determined by comparison with the standard curve.

### mRNA extraction and qRT-PCR

TRIzol reagent was used to extract the total RNA from the lung tissues. RNA concentration was detected using absorbance values at 260 nm obtained on a NanoDrop 2000 (Thermo Fisher Scientific, USA) spectrophotometer. cDNA was prepared by reverse transcription according to the manufacturer's instructions and stored at -20°C until use. Real-time PCR was performed in triplicate using the SYBR Green master mix. The reaction conditions were 95°C for 30 s, 95°C for 5 s, and 40 cycles of 60°C for 34 s. The relative change in gene expression was calculated using 2^-ΔΔCT^ and GAPDH as an internal reference gene. The following primer pairs were used: GAPDH, upstream primer 5′-TGTGTCCGTCGTGGATCTGA-3′, downstream primer 5′-TTGCTGTTGAAGTCGCAGGAG-3′; Jagged1, upstream primer 5′-CCAGCGGTCCTAATGGTGATG-3′, downstream primer 5′-GCTGTGGTTCTGAGCTGCAAAG-3′.

### Flow cytometry analysis

The mouse lung tissue was digested in 2 mL of RPMI 1640 medium (Gibco, USA) containing collagenase IV (2.5 g/L) at 37°C for 40 min. The cell suspension and the incompletely digested lung and spleen tissue pellet were then ground using a 200 µm mesh filter, centrifuged at 250 *g* for 5 min at 4^o^C. The supernatant was discarded, and the pellet was incubated in red blood cell lysis buffer for 4 min in the dark and then centrifuged (250 *g* for 5 min at 4^o^C). The supernatant was discarded again, and the pellet was washed with PBS. The magnetic bead cells were separated and cultured in a cell incubator at 5% CO_2_ and 37°C for 48 h. The retained pellet containing lung mononuclear cells was resuspended at 10^6^ cells/mL in 1 mL of RPMI 1640 medium containing 10% fetal bovine serum, 25 μg/L of PMA, 1 μg/L ionomycin, and 0.2% monensin. The cell suspension was incubated for 4 h at 5% CO_2_ and 37°C and then centrifuged (250 *g* for 5 min at 4^o^C). Percp-Cy5-5 anti-CD3 antibody and APC anti-γδT17 antibody were added to the pellet and incubated at 4°C for 30 min in the dark. The cells were then washed with PBS, resuspended in Cytofix/Cytoperm solution (Becton Dickinson, USA), and incubated for 20 min at 4°C in the dark. After that, the cells were rinsed with washing buffer and incubated with PE anti-IL-17 antibody for 30 min, then washed twice with PBS, and resuspended in 200 μL of PBS. Flow cytometry analysis was performed using FlowJo 7.6 software (Becton Dickinson).

### Statistical analysis

Statistical analysis was performed using the SPSS 22.0 software (IBM, USA) and the graphics were generated using the Prism 5.0 software (GraphPad, USA). The data are reported as means±SE. ANOVA was used to analyze the differences among group means, followed by post-hoc Fisher's least significant difference (LSD) test for pairwise comparisons between groups. Pearson correlation was used to measure the correlation between samples. A P-value <0.05 was considered statistically significant.

## Results

### Mouse airway reactivity

**Figure 1:**
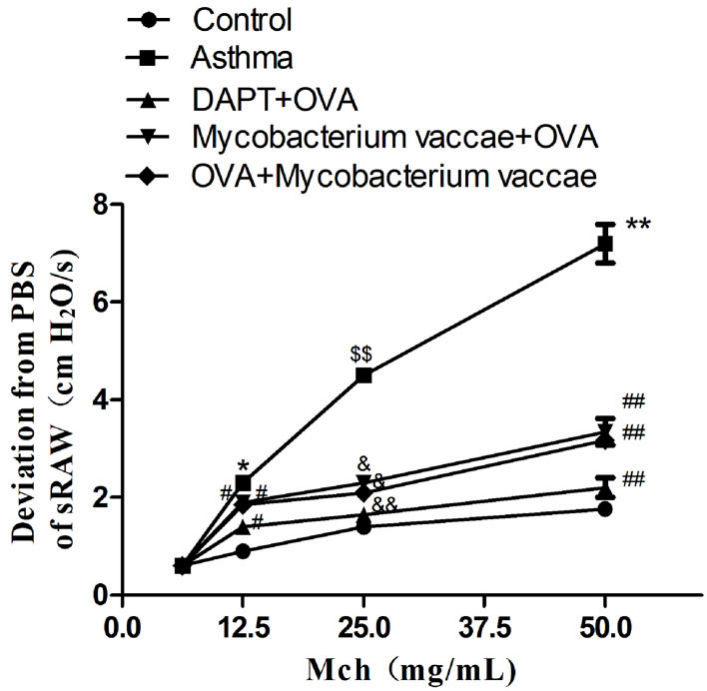
Effects of inhaled inactivated *Mycobacterium vaccae* on airway hyperresponsiveness with methacholine (Mch) treatment. Airway reactivity was measured using specific airway resistance (sRaw). Ovalbumin (OVA) challenge significantly increased sRaw in all 4 methacholine doses with the maximum increase achieved in the dose 50 mg/mL. Data are reported as mean±SE relative percentage to the control group (n=8). *P<0.05, ^$$^P<0.01, **P<0.01 *vs* control group; ^#^P<0.05, ^&^P<0.05, ^&&^P<0.01, ^##^P<0.01 *vs* asthma group (ANOVA).

Figure 1 shows the airway reactivity in the five experimental groups. The airway resistance (sRaw values) of the mice in the asthma group was significantly higher than that of the control group after stimulation with 12.5, 25, and 50 mg/mL of Mch (P<0.05). The difference between the control group and the blocking, prevention, and treatment groups was also statistically significant (P<0.01). There was no significant difference in airway reactivity among all groups after 6.25 mg/mL Mch stimulation (P>0.05).

### Lung histopathology

**Figure 2:**
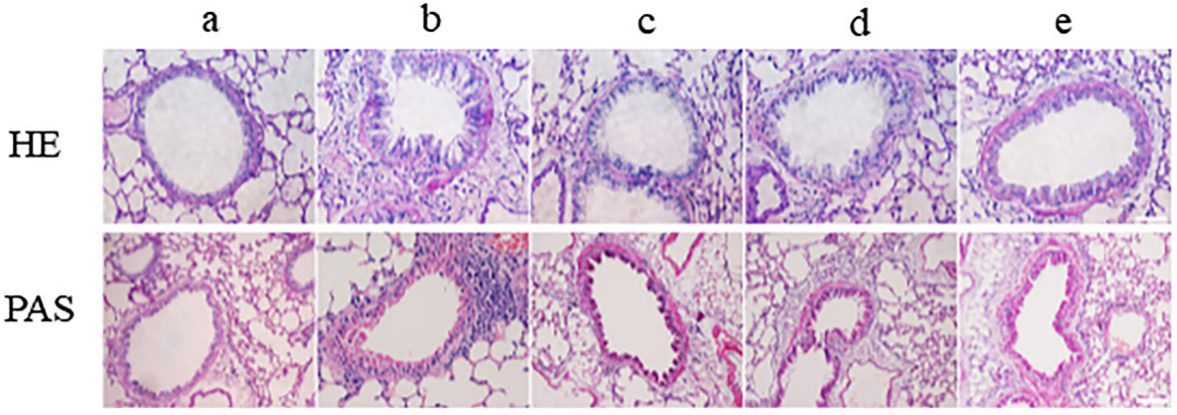
Lung histopathology was detected using hematoxylin and eosin (HE) and Periodic acid-Schiff (PAS) staining. Effects of inactivated *Mycobacterium vaccae* on ovalbumin (OVA)-induced airway inflammation. Histological examination of normal control group (**a**), asthma group (**b**), gamma-secretase inhibitor (DAPT + OVA group (**c**), *M. vaccae* + OVA group (**d**), OVA + *M. vaccae* group (**e**). HE staining: original magnification ×400, scale bar=50 μm; PAS staining: original magnification ×200, scale bar=100 μm.

Lung HE and PAS staining showed that the control group had normal bronchial morphology, no hyperplasia of epithelial cells, no thickening of the walls, normal alveolar septum, and few inflammatory cells around the bronchi and blood vessels. PAS staining of control lungs showed a very small amount of goblet cells in the airway epithelium and no mucus oozing. Compared to the control lungs, the asthma group lungs showed greater inflammatory cells infiltration around the bronchus and blood vessels, in addition to bronchial lumen stenosis and thickened walls. PAS staining of the asthma group showed goblet cells hyperplasia and more prominent mucus oozing. In comparison with the asthma group, the bronchial and perivascular inflammatory cells infiltration, as well as goblet cells and mucus oozing, were significantly reduced in the blocking, prevention, and treatment groups (Figure 2).

### Immunohistochemistry and immunofluorescence

**Figure 3:**
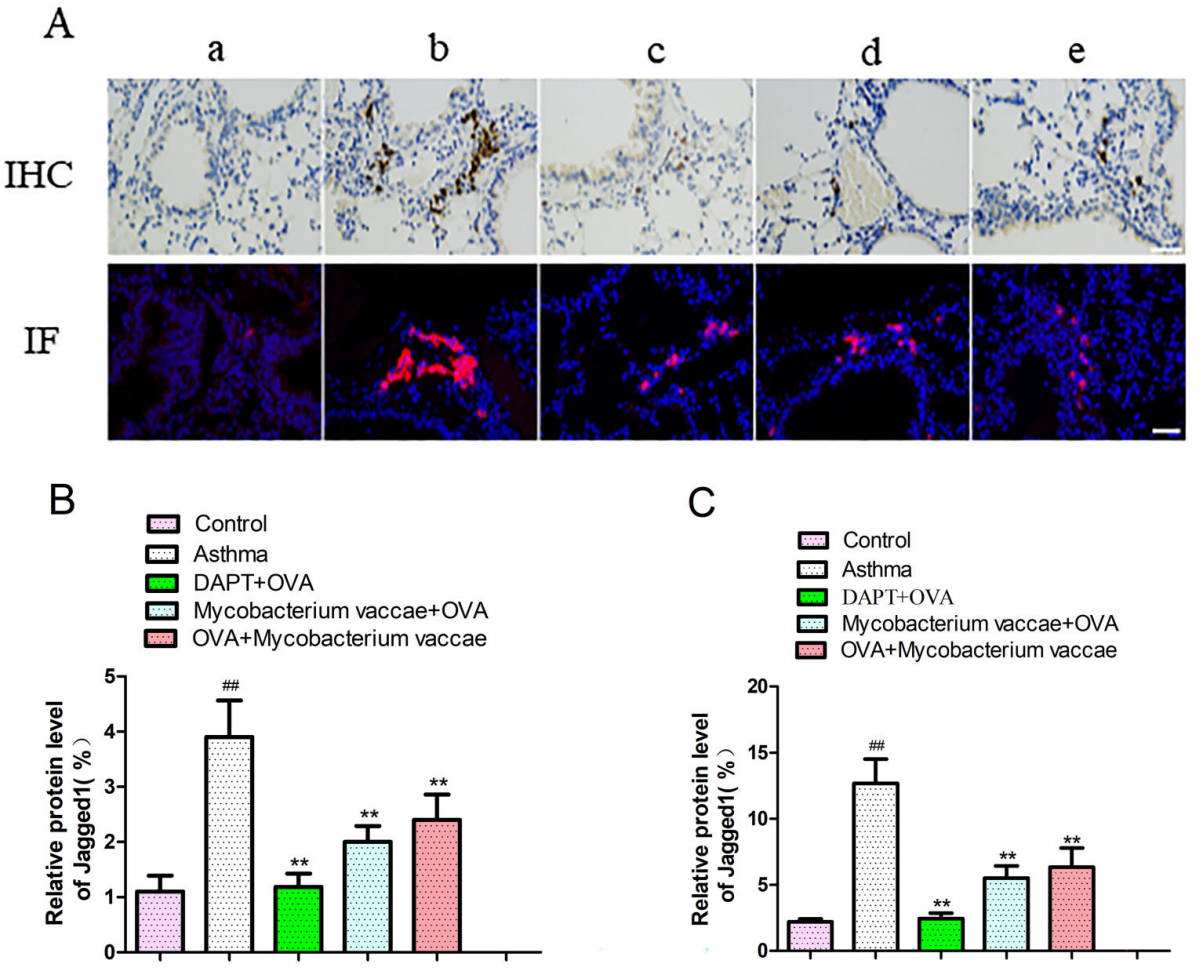
Expression of Jagged1 using immunohistochemical and immunofluorescence stains (**A**) in mice in control group (**a**), asthma group (**b**), gamma-secretase inhibitor (DAPT) + ovalbumin (OVA) group (**c**), *Mycobacterium vaccae* + OVA group (**d**), OVA + *M. vaccae* group (**e**), and its quantification (**B** and **C**). Data are reported as mean±SE relative percentage to the control group (n=8). ^##^P<0.01 *vs* control; **P <0.01 *vs* asthma group (ANVOA). Original magnification x400, scale bar=50 μm.

The expression of Jagged1 in the lung tissue of asthmatic mice increased significantly compared to the control group (P<0.05). Interestingly, the expression of Jagged1 was decreased in the lung tissue of the blocking, prevention, and treatment groups. The difference was statistically significant in the three groups, compared to the asthma group (Figure 3, P<0.05).

### Jagged1 mRNA expression in mouse lung tissues

**Figure 4:**
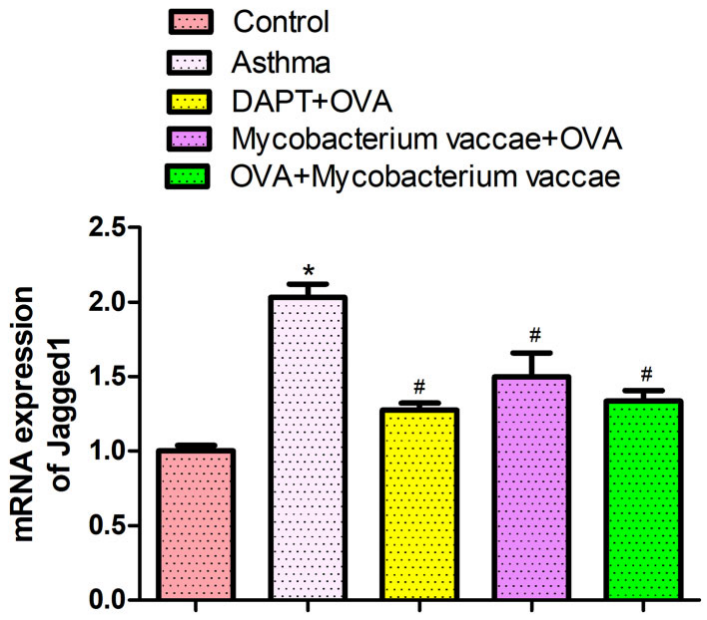
The mRNA expression of Jagged1 in lung tissues was determined using real-time PCR. Data are reported as mean±SE relative percentage to the control group (n=8). *P<0.05 *vs* control; ^#^P<0.05 *vs* asthma group (ANOVA). OVA: ovalbumin; DAPT: gamma-secretase inhibitor.

The mRNA expression of Jagged1 in the asthma group was significantly greater than in the normal group (P<0.01). Importantly, the expression of Jagged1 mRNA in the prevention and treatment groups was significantly lower than that in the asthma group (Figure 4, P<0.05).

### IL-17A levels in the serum

**Figure 5:**
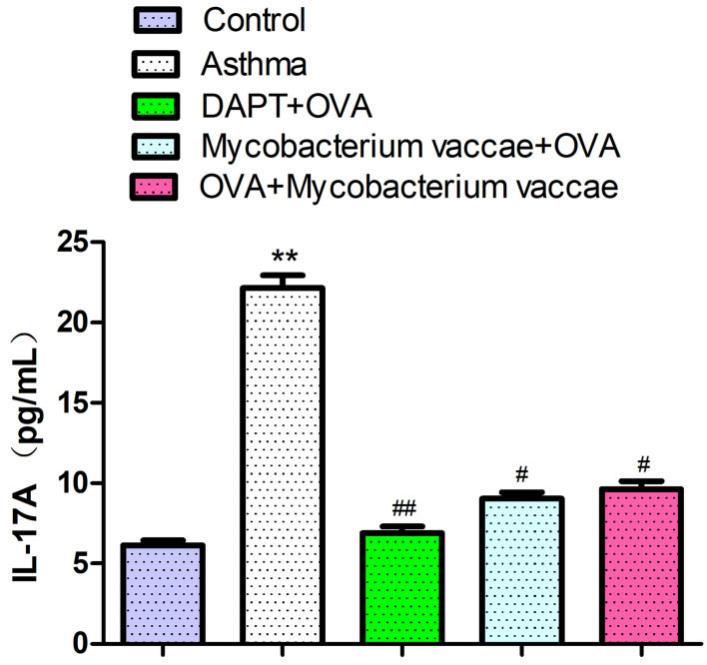
Interleukin (IL)-17A concentrations in serum by ELISA using specific cytokine detection kits. Data are reported as mean±SE relative percentage to the control group (n=8). **P<0.01 *vs* control; ^#^P<0.05, ^##^P<0.01 *vs* asthma group (ANOVA). OVA: ovalbumin; DAPT: gamma-secretase inhibitor.

IL-17A levels in the serum were significantly higher in the asthma group, compared to the normal group (P<0.05). In addition, the IL-7A level in the serum of the prevention, treatment, and blocking groups was significantly lower than in the asthma groups (Figure 5, P<0.05).

### Correlation analysis between Jagged1 and γδT17 cells in the lung tissues

**Figure 6:**
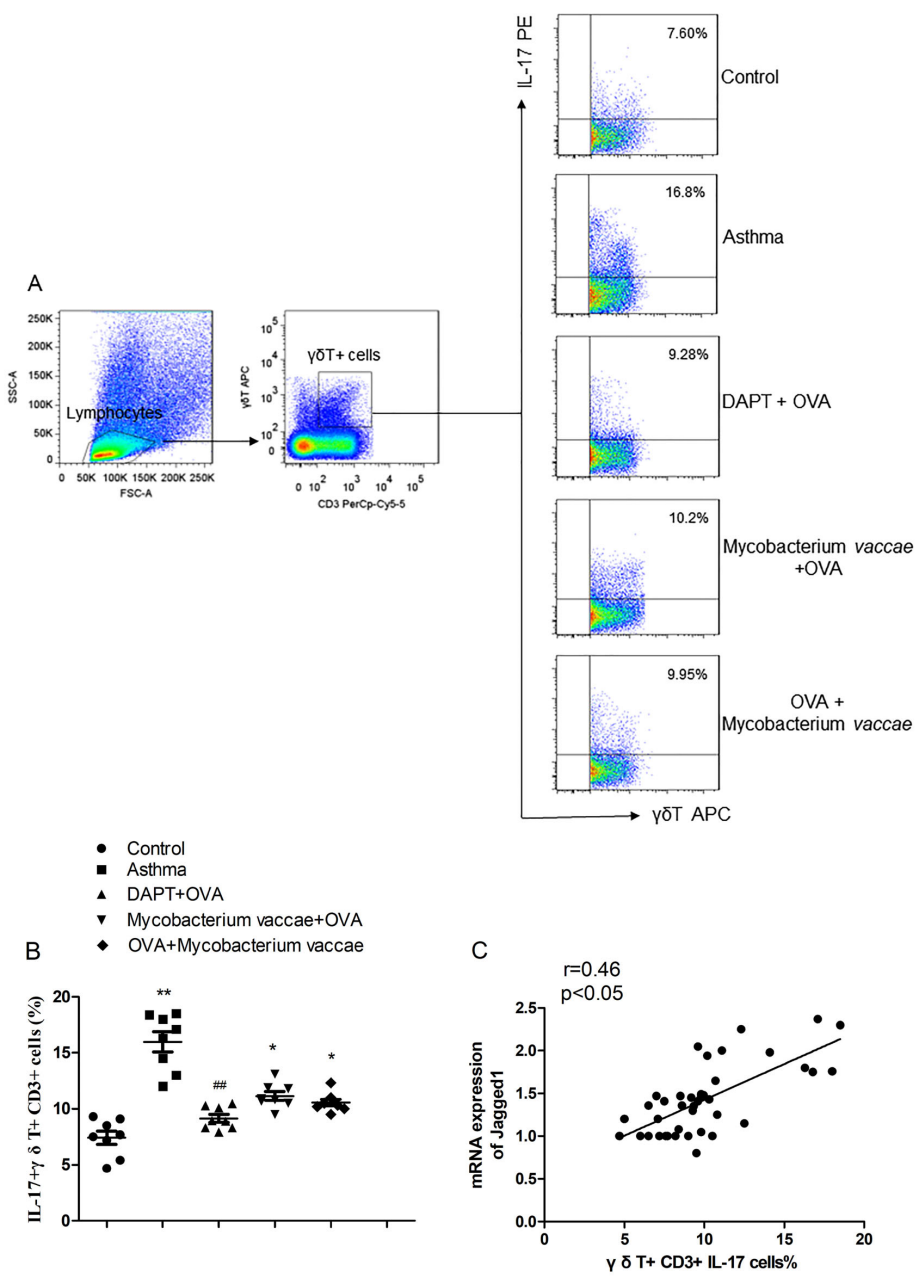
Correlation analysis between Jagged1 and γδT17 cells in lung tissues. **A** and **B**, The ratio of IL-17+γδT+ cells to T cells was measured in each group. **C**, The percentage of IL-17+γδT+ cells and the Jagged1 mRNA expression in lung tissues were positively correlated (r=0.46, P<0.05). Data are reported as mean±SE relative percentage to the control group (n=8). **P<0.01 *vs* control; *P<0.05, ^##^P<0.01 *vs* asthma group (ANOVA). OVA: ovalbumin; DAPT: gamma-secretase inhibitor.

**Figure 7:**
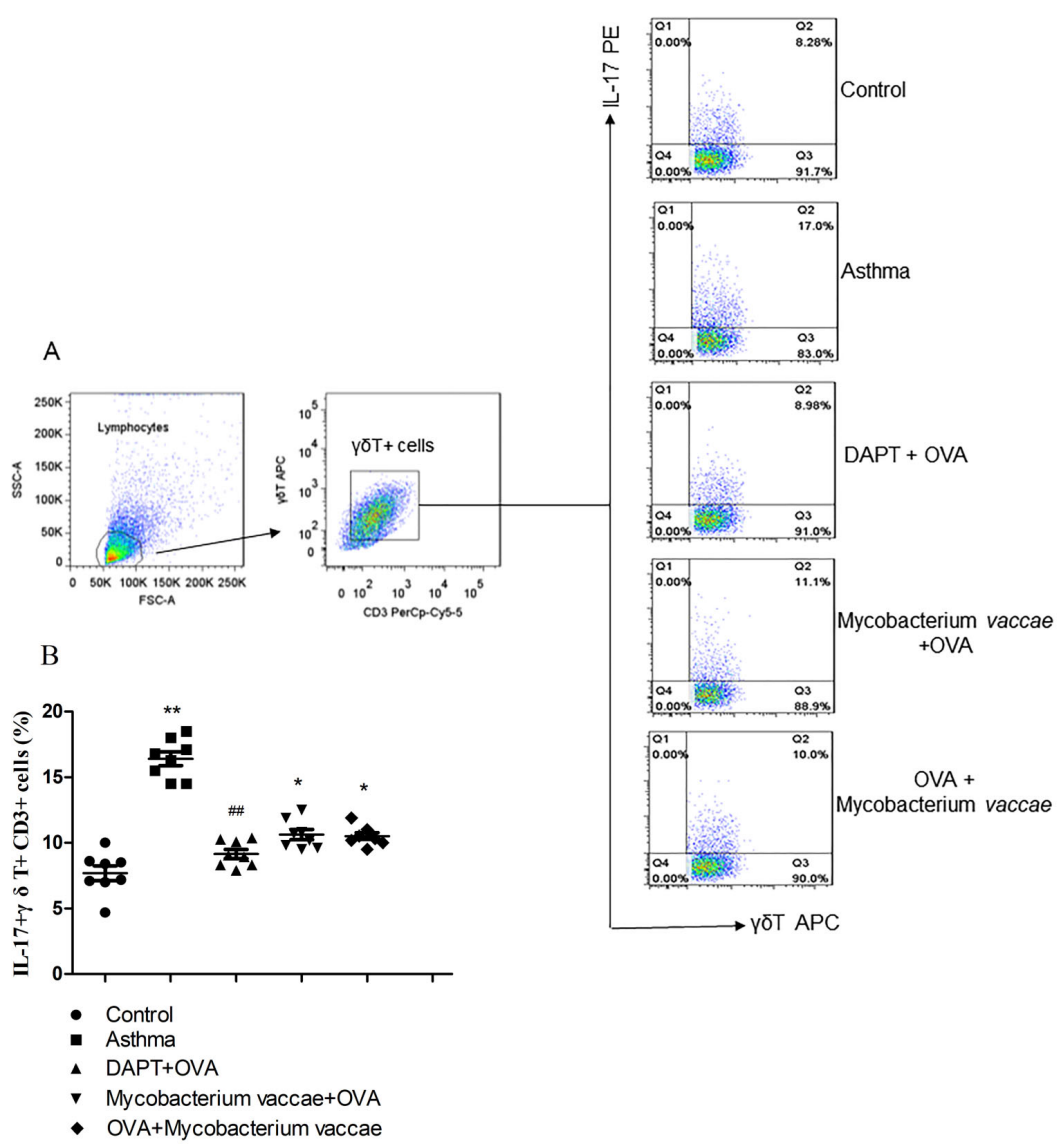
*Mycobacterium vaccae* spleens from ovalbumin (OVA)-treated mice were compared to the control, asthma, gamma-secretase inhibitor (DAPT)+OVA, *M. vaccae* + OVA, and OVA + *M. vaccae* groups to determine the percentage of γδT17 cells among the CD3+ T cells by flow cytometry. Data are reported as mean±SE relative percentage to the control group (n=8). **P<0.01 *vs* control; *P<0.05, ^##^P<0.01 *vs* asthma group (ANOVA).

The level of IL-17+ of γδT17 cells in the lung tissues from the asthma group was higher than that of the control group (P<0.05). Interestingly, compared to the asthma group, the ratio of IL-17+γδT+ cells to T cells was lower in the prevention, treatment, and blocking groups (Figures 6 and 7, P<0.05 for each group). The percentage of IL-17+γδT+ cells and the mRNA expression of Jagged1 in the lung tissues was positively correlated (r=0.46, P<0.05). This may suggest that the Notch signaling pathway had a regulatory effect on γδT17 cells in asthmatic mice and that inhalation of inactivated *M. vaccae* can regulate γδT17 secretion in asthmatic mice.

## Discussion

IL-17 has a powerful role in recruiting and activating neutrophils, especially in patients with severe asthma and glucocorticoid resistance. IL-17 can aggravate airway inflammatory cell infiltration and promote airway remodeling and hyperresponsiveness. IL-17 acts directly on epithelial cells to promote goblet cells hyperplasia and mucus gland secretion (22), which in turn promotes pulmonary fibrosis by inducing TGF-β and collagen expression (23). γδT cells show an imbalance in Th1/Th2 ratio and evidence suggests that γδT cells are involved in the development of asthma (4,24). γδT cells and memory αβT cells produce similar levels of IL-17 (25). γδT17 cells induce the production of inflammatory cytokines, promote the release of inflammatory transmitters, and participate in the development of inflammatory reactions. In our study, we showed the role of the Notch signaling pathway in asthma using the Notch inhibitor DAPT. Our results showed that, although IL-17 cytokine expression increased significantly in asthma, the inhibition of Notch signaling decreased airway responsiveness and inflammation in asthmatic mice. In addition, the expression of cytokine IL-17 in γδT17 cells increased significantly in the lungs of asthmatic mice. Interestingly, Jagged1 mRNA expression was positively correlated with the percentage of CD3+/γδT+ cells and IL-17+γδT+ cells in the lungs of mice. This suggested that the expression level of Jagged1 is closely related to the pathogenesis of asthma. Furthermore, we showed that the expression of γδT17 cell-associated cytokine IL-17 in the spleen of mice decreased significantly in the prevention and treatment groups that used *M. vaccae*. These findings indicated that OVA and *M. vaccae* may affect the percentage of γδT17 cells in the spleen of animals. Moreover, this may also suggest that the Notch signaling pathway participated in the pathogenesis of asthma by regulating the secretion of IL-17 by γδT17 cells through Jagged1 receptor.

We have shown in previous studies that γδT17 cells are involved in the pathogenesis of asthma induction (26), which was confirmed by the presence of an imbalance in the Th1/Th2 ratio of γδT cells in asthma animal models (27). Inhalation of *M. phlei* has been shown to correct the Th1/Th2 ratio in asthma (12,13) and reverse the Th1/Th2 ratio of γδT cells thereby reducing lung inflammation. *M. vaccae* and *M. phlei* belong to the genus Mycobacterium and show similar immunomodulatory effects. Aerosol inhalation can stimulate the cellular immune response of the body through mucosal immunity to regulate gas, which can lead to airway inflammation and hyperresponsiveness. The present experimental study showed that both co-treatment and post-treatment with *M. vaccae* inhalation can reduce airway responsiveness and inflammation and inhibit γδT17 cells differentiation and IL-17 secretion. Correlation analysis between the prevention and the treatment groups showed that Jagged1 mRNA expression was correlated with γδT17 cells percentage, which suggested that atomized cow-related Mycobacterium can inhibit the production of γδT17 cells.

Many signaling pathways have been reported to be involved in the pathogenesis of asthma, including the Notch signaling pathway. This is an evolutionary conserved signaling pathway that plays key roles in the regulation of cell differentiation, development, proliferation, and survival (28). Although delta ligands are associated with the development of Th1 cells, Jagged ligands contribute to the development of Th2 cells. The main features of asthma are Th2 induction, Th1 cells reduction, and imbalance in Th1/Th2 ratio, in addition to excessive mucin secretion, airway inflammation, hyperresponsiveness, and remodeling. Many studies have shown that Notch signaling pathway can induce Th2-type cell differentiation and participate in the pathogenesis of asthma (29-32). When the body is infected by allergens, such as bacteria and viruses, it triggers a specific immune response through the antigen-presenting cells (APC). These cells present information to peripheral T cells and induce peripheral CD4+ T cells to differentiate into the Th1 subtype. T cell signaling is accomplished by the Notch signaling pathway, which plays key roles in directing Th1 and Th2 differentiation (33). Amsen et al. (28) reviewed evidence suggesting that the Notch pathway can mediate signals, and how Notch instructs Th-cell differentiation during normal and pathological immune responses. The Notch signaling pathway has been shown to be involved in the pathogenesis of allergic airway inflammation and is considered a new therapeutic target for asthma patients (32). Recent studies have reported that Jagged1 can lead to increased Th cell differentiation and is involved in airway inflammatory responses (18,19). Indeed, studies have shown that silencing Jagged1 expression in asthma can reduce airway allergic reactions, inhibit Th2 cytokine secretion, and thus correct the imbalance in the Th1/Th2 cell ratio (34).

In conclusion, our results showed that nebulization of *M. vaccae* can reduce airway inflammation in asthmatic mice by regulating γδT17-based IL-17 secretion through the downregulation of Jagged1. The regulation of γδT17 by Jagged bacillus may be one of the target pathways for new asthma treatments. However, these results were obtained from animal models and tests in humans must be performed to validate them.
